# Impact of panel design and cut-off on tumour mutational burden assessment in metastatic solid tumour samples

**DOI:** 10.1038/s41416-020-0762-5

**Published:** 2020-02-25

**Authors:** Joanne M. Mankor, Marthe S. Paats, Floris H. Groenendijk, Paul Roepman, Winand N. M. Dinjens, Hendrikus J. Dubbink, Stefan Sleijfer, CPCT Consortium, Edwin Cuppen, Martijn P. J. K. Lolkema

**Affiliations:** 1 Department of Pulmonary Medicine, Erasmus MC, Rotterdam, The Netherlands; 2 Department of Pathology, Erasmus MC, Rotterdam, The Netherlands; 3 Hartwig Medical Foundation, Amsterdam, The Netherlands; 4 Department of Medical Oncology, Erasmus MC Cancer Institute, Erasmus MC, Rotterdam, The Netherlands; 5 Center for Personalized Cancer Treatment, Amsterdam, The Netherlands; 6 Center for Molecular Medicine and Oncode Institute, University Medical Center, Utrecht, The Netherlands

**Keywords:** Molecular medicine, Cancer genetics, Predictive markers, Molecular medicine, Cancer genetics

## Abstract

Tumour mutational burden (TMB) has emerged as a promising biomarker to predict immune checkpoint inhibitors (ICIs) response in advanced solid cancers. However, harmonisation of TMB reporting by targeted gene panels is lacking, especially in metastatic tumour samples. To address this issue, we used data of 2841 whole-genome sequenced metastatic cancer biopsies to perform an in silico analysis of TMB determined by seven gene panels (FD1CDx, MSK-IMPACT™, Caris Molecular Intelligence, Tempus xT, Oncomine Tumour Mutation Load, NeoTYPE Discovery Profile and CANCERPLEX) compared to exome-based TMB as a golden standard. Misclassification rates declined from up to 30% to <1% when the cut-point for high TMB was increased. Receiver operating characteristic analysis demonstrated that, for correct classification, the cut-point for each gene panel may vary more than 20%. In conclusion, we here demonstrate that a major limitation for the use of gene panels is inter-assay variation and the need for dynamic thresholds to compare TMB outcomes.

## Background

Tumour mutational burden (TMB) has emerged as a promising biomarker to predict response to immune checkpoint inhibitors (ICIs) in a number of solid cancers. The reason TMB was proposed as predictive biomarker is the assumption that more mutations would possibly generate a higher number of new epitopes, called “neoantigens”, also referred to as mutational load (ML). A higher ML increases the probability that a tumour cell expresses a true neoantigen rendering a tumour cell more prone to T cell-mediated immune destruction.^1,2^

TMB is defined as the number of mutations (somatic single variant (SNV) and multinucleotide variant (MNV) and small insertions and deletions (indels)) per megabase pair (Mb) of sequence examined and can be measured by genome, exome or gene panel sequencing. This introduces a challenge because mutations are not randomly distributed throughout the genome and therefore the sequencing design will introduce a bias.^3^ Since whole-exome sequencing or whole-genome sequencing (WGS) techniques are not yet routinely used in clinical practice, panel-based sequencing methods have become feasible alternatives ready to be implemented in routine diagnostics. Still, how gene panel-based TMB relates to exome-based TMB and whether outcomes of different gene panel platforms are translatable, remains unknown. Budczies et al.^4^ recently described the limitations of panel-based TMB measurements by simulating TMB in publicly available datasets of primary tumours. However, effectiveness of ICI treatment is primarily described in metastatic disease and all Food Drug Administration approvals so far are granted to ICIs for the treatment of advanced stage disease. Since we know that mutational patterns may differ between primary tumour and metastases, selection of patients for ICI treatment is ideally based on biomarker detection in samples from metastatic tumours.^5–7^ Therefore, analysis of the concordance between different panels on metastatic samples is a relevant analysis.

## Methods

Since a comprehensive comparison between available targeted gene panels and exome- or genome-based TMB in metastatic tumour samples is lacking, we here assessed the variety of TMB measured by seven different panels based on WGS as a reference. We used TMB based on exome as a reference standard because the coding sequence is most frequently examined in the context of TMB. We hypothesised that classifying patients in two categories of high versus intermediate or low TMB by seven different gene panels would not necessarily result in the same outcome as panel sizes and gene content differ and typically only a limited number of mutations are measured in these assays. By using data of 2841 whole-genome-sequenced metastatic cancer biopsies^8^ as a reference, we performed an in silico analysis of TMB determined by seven gene panels (FD1CDx by Foundation Medicine, MSK-IMPACT™ by Memorial Sloan Cancer Centre, Caris Molecular Intelligence by Caris Life Sciences, Tempus xT by Tempus, Oncomine Tumour Mutation Load by ThermoFisher, NeoTYPE Discovery Profile by NeoGenomics and CANCERPLEX by KEW) compared to exome TMB as a golden standard. For TMB determination, the number of variants (SNVs, MNVs and indels) within the panel design were divided by the panel footprint (0.78–1.48 Mb) or the size of the exome (30 Mb). Panel designs were retrieved from the manufacturer’s website and as exact designs are not available, panel footprint was assumed to encompass the longest open reading frame of each gene as based on Ensembl (GRCh38), although the real panel design will in most cases be smaller.

## Results

**Fig. 1:**
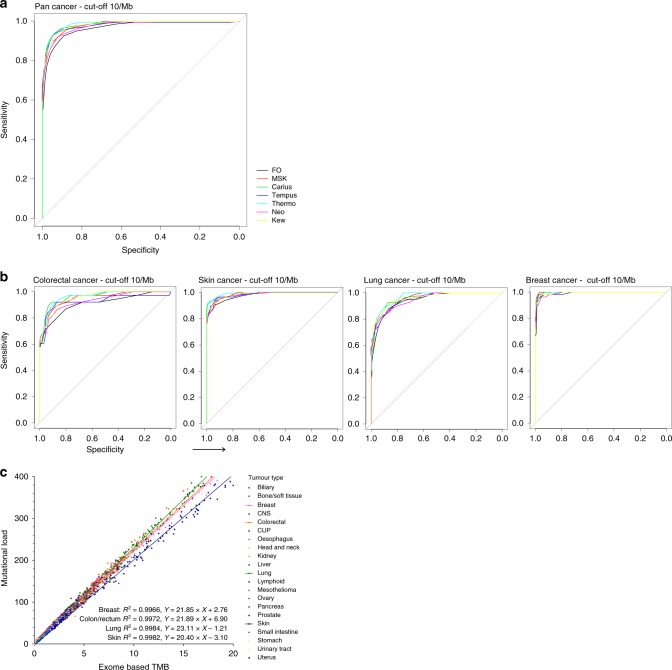
Performance of the 7 different gene panels in exome-based TMB determination and the relation between TMB and ML for different types of cancer. **a** Receiver operating characteristic (ROC) curves for each gene panel compared to exome-based TMB for all tumour types in the cohort. Exome-based TMB was dichotomised at a 10/Mb cut-point. **b** ROC curves for each gene panel compared to exome-based TMB for colorectal cancer, skin cancer, lung cancer and breast cancer, respectively. Exome-based TMB was dichotomised at a 10/Mb cut-point. **c** For each tumour biopsy sequenced, mutational load is plotted against exome-based TMB. Linear regression lines are fitted on the colorectal cancer, skin cancer, lung cancer and breast cancer datasets, respectively. Goodness of fit (*R*^2^) and equations for the regression lines of these four tumour types are depicted in the graph.

First, we analysed how patients would have been classified by each gene panel compared to exome-based TMB for different TMB cut-offs. As expected, the misclassification rate (sum of the percentages of false positives and false negatives) declines from up to 30% to <1% when the cut-off is increased from 5 to 40 mutations per Mb (Table 1). The high percentages of false positives at lower thresholds of high TMB indicate that panels are generally over-calling. This is most certainly due to the fact that panels consist of oncogenic driver genes that will have a disproportionate effect on the mutation count. Second, we dichotomised our data with a cut-point of 10 mutations per Mb to define high TMB, as this cut-off is most frequently used in recent trials as a threshold for high TMB.^9–11^ At this cut-off, misclassification rates range between 4 and 10%, with largest panels typically performing best. Next, we performed a receiver operating characteristic (ROC) analysis for the different gene panels to determine the threshold that each gene panel should set in order to classify most patients in the right TMB category compared to a 10/Mb exome-based cut-off for high TMB (Fig. 1a). By adjusting the thresholds for each panel, a high correct pan-cancer classification of patients could be obtained, with area under the curves (AUCs) ranging from 0.97 to 0.98. However, larger differences (AUC 0.911–0.998) appeared when panel reliability was assessed for different tumour types (Fig. 1b), which is likely due to tumour-type-specific differences in TMB distribution.

**Table 1:** TMB determined by variants (SNVs, MNVs and indels) in targeted genes in various gene panels compared to TMB determined by the measurement of all variants (SNVs, MNVs and indels) in the exome.

Panel			Cut-off 5/Mb		Misclassified	Cut-off 10/Mb		Misclassified	Cut-off 20/Mb		Misclassified	Cut-off 40/Mb		Misclassified
FoundationOne	TP	FN	29.9%	2.2%	29.2%	12.9%	1.1%	10.3%	5.9%	0.7%	2.1%	2.4%	0.6%	0.9%
	FP	TN	27.0%	40.9%		9.3%	76.7%		1.4%	91.9%		0.3%	96.7%	
MSK-IMPACT	TP	FN	29.8%	2.0%	18.1%	12.5%	1.4%	4.9%	5.8%	0.6%	1.2%	2.5%	0.4%	0.7%
	FP	TN	16.1%	49.3%		3.6%	81.8%		0.5%	92.8%		0.2%	96.7%	
Caris	TP	FN	28.8%	2.8%	12.9%	12.6%	1.3%	3.8%	5.5%	0.9%	1.3%	2.2%	0.7%	0.8%
	FP	TN	10.1%	56.3%		2.5%	83.0%		0.4%	92.9%		0.1%	96.9%	
Tempus xT	TP	FN	29.6%	2.2%	13.5%	12.6%	1.3%	4.2%	5.8%	0.7%	1.2%	2.6%	0.3%	0.5%
	FP	TN	11.3%	54.9%		2.9%	82.6%		0.5%	92.8%		0.2%	96.7%	
ThermoFisher	TP	FN	29.3%	2.4%	14.6%	13.1%	0.9%	4.9%	5.9%	0.5%	1.4%	2.8%	0.2%	0.4%
	FP	TN	12.2%	53.8%		4.0%	81.3%		0.9%	92.4%		0.2%	96.7%	
NeoGenomics	TP	FN	29.9%	1.9%	22.2%	12.9%	1.0%	6.8%	6.1%	0.4%	1.6%	2.7%	0.2%	0.7%
	FP	TN	20.4%	44.4%		5.8%	79.2%		1.1%	92.1%		0.4%	96.5%	
Cancerplex	TP	FN	29.1%	2.6%	15.2%	12.5%	1.3%	5.0%	5.8%	0.7%	1.5%	2.6%	0.4%	0.6%
	FP	TN	12.6%	53.4%		3.7%	81.6%		0.8%	92.4%		0.2%	96.7%	

*TMB* tumour mutational burden, *TP* true positive, *FP* false positive, *TN* true negative, *FN* false negative.

The number of patients classified as TP, FP, TN and FN for each cut-off for high TMB (5, 10, 20 and 40/Mb) is depicted. The percentage “misclassified” for each cut-off and panel is the sum of the percentages FP and FN.

## Discussion

Here we show that, because of design differences, it is crucial to adjust the cut-off for each panel design as, for example, for correct classification at 10 mutations per Mb this may vary more than 20% (from 7.8 to 11.7) between commonly used test panels (Supplementary Table 1). It should be noted that in practice differences might even be larger due to experimental and variant calling differences between platforms. Nevertheless, a major limitation for the use of targeted sequencing platforms is the inter-assay variation due to design and the need for dynamic thresholds to compare TMB outcomes—for different platforms and cancer types. Specifically, trials that use TMB determined by a specific panel as a prospective selection biomarker can only result in platform- and disease-specific patient selection.

Several other factors impact the reliability of the number of mutations counted in the tumour genome. First, it should be realised that TMB does not reflect the number of neoantigens in a tumour cell that can be acted upon by the immune system. ML, the total number of non-synonymous SNVs, MNVs and indels in the tumour, would actually be a more relevant measurement because these mutations result (theoretically) in a change in amino acid(s) and may thus lead to potential neoantigens. Interestingly, ML and TMB do have a clear linear relationship, but in a tumour-type-specific manner. For example, a TMB of 10 mutations per Mb corresponds to a ML of ~20 × 10 in skin cancer and ~23 × 10 in lung cancer (Fig. 1c).

Second, this in silico analysis does not take into account the quality of the sample used for sequencing. In the studied cohort, WGS was performed on fresh frozen tumour tissue, but in daily clinical practice, formalin-fixed, paraffin-embedded tumour tissue is primarily used as a template for targeted sequencing.

Third, matching blood samples for determination of germ line variants are crucial for a valuable TMB assessment in both WGS and gene panel sequencing, since germline polymorphisms can easily contaminate the mutation count.^12^ Most gene panel platforms use tumour-only sequencing and filter germline variants out by using large germline variant datasets.

In conclusion, we would like to underscore the importance of whole-exome- or whole-genome-based mutation measurements of metastatic tumour samples for benchmarking TMB-based diagnostic biomarker platforms. Both of these platforms do potentially detect all mutations and all coding mutations, respectively, and lack the variability that comes with panel-based diagnostics. Thus, comprehensive tumour sequencing would be the most optimal strategy for the development of tumour-type agnostic, reproducible and reliable genetic biomarkers for immunotherapy.

## Supplementary information

Supplementary table 1

## Data Availability

All data described in this study is freely available from the Hartwig Medical Foundation for academic research within the constraints of the consent given by the patients. Standardised procedures and request forms can be found at https://www.hartwigmedicalfoundation.nl/en. All bioinformatic analysis tools and scripts used are available at https://github.com/hartwigmedical/.
