# Supplementary material for: Impact of panel design and cut-off on tumour mutational burden assessment in metastatic solid tumour samples
Source: Br J Cancer. 2020 Feb 25;122(7):953–6. doi: 10.1038/s41416-020-0762-5 (PMC7109082; doi:10.1038/s41416-020-0762-5)
Supplement: Supplementary file 1 — Supplementary table 1 [file 41416_2020_762_MOESM1_ESM.docx]

Supplementary methods

A detailed description of the methods used for whole-genome sequencing and variant calling can be found in the paper by Priestley et al (1). In short, core needle biopsies from metastatic lesions were taken from patients with advanced cancer that consented to participation in the CPCT-02 (NCT01855477) and DRUP (NCT02925234) clinical studies. Only samples with at least 20% tumor cell purity were included in the analyses. Sequencing depths were on average 106x and 38x for tumor tissue and normal tissue (blood samples) respectively. Variant calling for somatic SNV and indels was performed using Strelka v. 1.0.14 (2) with optimized settings and post-calling filtering.

References

1. Priestley P, Baber J, Lolkema MP, Steeghs N, de Bruijn E, Shale C, et al. Pan-cancer whole-genome analyses of metastatic solid tumours. Nature. 2019;575(7781):210-6.

2. Saunders CT, Wong WS, Swamy S, Becq J, Murray LJ, Cheetham RK. Strelka: accurate somatic small-variant calling from sequenced tumor-normal sample pairs. Bioinformatics. 2012;28(14):1811-7.

**Supplementary table 1**

Threshold, specificitity, sensitivity and area under the curve (AUC) derived from the receiver operating characteristics analysis (ROC) for all tumor types in the cohort and for the four different cancer types.

**Pan cancer, exome TMB, cut-off 10/Mb: n=2841**

|  | threshold | specificity | sensitivity | AUC |
| --- | --- | --- | --- | --- |
| FO | 9,581 | 0,892 | 0,927 | 0,97 |
| MSK | 9,978 | 0,952 | 0,891 | 0,982 |
| Caris | 8,443 | 0,936 | 0,942 | 0,985 |
| Tempus | 9,188 | 0,946 | 0,929 | 0,981 |
| Thermo | 10,089 | 0,945 | 0,929 | 0,987 |
| Neo | 10,435 | 0,922 | 0,922 | 0,978 |
| Kew | 9,317 | 0,928 | 0,929 | 0,983 |

**Breast cancer, exome TMB, cut-off 10/Mb: n=532**

|  | threshold | specificity | sensitivity | AUC |
| --- | --- | --- | --- | --- |
| FO | 10,858 | 0,96 | 0,962 | 0,992 |
| MSK | 9,978 | 0,969 | 1,0000 | 0,998 |
| Caris | 9,793 | 0,975 | 0,981 | 0,997 |
| Tempus | 9,188 | 0,973 | 0,981 | 0,994 |
| Thermo | 8,475 | 0,95 | 0,981 | 0,996 |
| Neo | 10,435 | 0,954 | 0,962 | 0,992 |
| Kew | 9,317 | 0,956 | 0,981 | 0,995 |

**Colorectal cancer, exome TMB, cut-off 10/Mb: n=419**

|  | threshold | specificity | sensitivity | AUC |
| --- | --- | --- | --- | --- |
| FO | 10,858 | 0,801 | 0,868 | 0,911 |
| MSK | 9,978 | 0,871 | 0,895 | 0,951 |
| Caris | 9,793 | 0,906 | 0,921 | 0,964 |
| Tempus | 9,188 | 0,869 | 0,921 | 0,929 |
| Thermo | 11,703 | 0,911 | 0,895 | 0,963 |
| Neo | 11,534 | 0,861 | 0,868 | 0,932 |
| Kew | 10,128 | 0,869 | 0,895 | 0,954 |

**Lung cancer, exome TMB, cut-off 10/Mb: n=226**

|  | threshold | specificity | sensitivity | AUC |
| --- | --- | --- | --- | --- |
| FO | 9,581 | 0,855 | 0,877 | 0,943 |
| MSK | 9,978 | 0,938 | 0,815 | 0,956 |
| Caris | 7,767 | 0,869 | 0,926 | 0,957 |
| Tempus | 8,508 | 0,869 | 0,877 | 0,95 |
| Thermo | 10,089 | 0,89 | 0,901 | 0,966 |
| Neo | 11,534 | 0,931 | 0,802 | 0,948 |
| Kew | 9,317 | 0,897 | 0,877 | 0,958 |

**Skin cancer, exome TMB, cut-off 10/Mb. n=237**

|  | threshold | specificity | sensitivity | AUC |
| --- | --- | --- | --- | --- |
| FO | 9,581 | 0,967 | 0,897 | 0,978 |
| MSK | 8,243 | 0,925 | 0,932 | 0,984 |
| Caris | 9,118 | 0,992 | 0,915 | 0,989 |
| Tempus | 8,508 | 0,917 | 0,957 | 0,982 |
| Thermo | 9,282 | 0,983 | 0,932 | 0,991 |
| Neo | 9,337 | 0,933 | 0,932 | 0,983 |
| Kew | 9,317 | 0,958 | 0,897 | 0,986 |
